# Community Structure and Multi-Modal Oscillations in Complex Networks

**DOI:** 10.1371/journal.pone.0075569

**Published:** 2013-10-10

**Authors:** Henry Dorrian, Jon Borresen, Martyn Amos

**Affiliations:** School of Computing, Mathematics and Digital Technology, Manchester Metropolitan University, Manchester, United Kingdom; Umeå University, Sweden

## Abstract

In many types of network, the relationship between structure and function is of great significance. We are particularly interested in community structures, which arise in a wide variety of domains. We apply a simple oscillator model to networks with community structures and show that waves of regular oscillation are caused by synchronised clusters of nodes. Moreover, we show that such global oscillations may arise as a direct result of network topology. We also observe that additional modes of oscillation (as detected through frequency analysis) occur in networks with additional levels of topological hierarchy and that such modes may be directly related to network structure. We apply the method in two specific domains (metabolic networks and metropolitan transport) demonstrating the robustness of our results when applied to real world systems. We conclude that (where the distribution of oscillator frequencies and the interactions between them are known to be unimodal) our observations may be applicable to the detection of underlying community structure in networks, shedding further light on the general relationship between structure and function in complex systems.

## Introduction

The problem of relating the structure of a network to the dynamical behaviour it supports is of significant interest in a large number of domains. Many different systems may be represented as networks of connected entities, from friends communicating via social media [1], [2], to groups of neurons [3] and chemical reactions [4]. The fundamental issue we address here is how to link the observed dynamics of a system to certain properties of its underlying network structure. The hope is that, by deepening our understanding of how particular types of network behave (in a global sense) over time, we may gain the ability to predict the behaviour of so-far unknown networks with similar structures. In addition, by studying the recurring features of complex networks from a number of different disciplines, we may gain a deeper, more over-arching theoretical understanding of network dynamics.

Early work in this area focused on the development of model systems, which were used to analytically study the onset of certain behaviours (such as oscillations) [5], [6] (see also [7], [8] for reviews). These model systems have been successfully applied in a number of different disciplines, including chemistry [9], ecology [10] and sociology [2]. Of particular interest are networks which possess some form of *community structure* [11]–[15]; for an overview of methods for determining such community structure, see [16]. These are generally characterised as having groups of nodes that are tightly knit (i.e. highly connected) with less dense connections existing between these groups [17]. Such structures are interesting because many ‘real world’ networks (e.g. social, biological, technological) are naturally partitioned into sets of loosely-connected ‘communities’, or ‘modules’ [18]–[22]. Moreover, we do not restrict ourselves to networks which are static (i.e. we consider the possibility that connections are added and removed and nodes update their state) since such structures capture the fact that links between individual nodes - and the properties of nodes - may change over time. Recent work [23] on community structure in dynamic networks has shown that allowing nodes to influence the state of other nodes facilitates the spontaneous emergence of dynamic equilibrium (that is, the community structure of the network remains stable, even as group composition changes over time) [24]. The idea of nodes influencing one another leads naturally to the notion of synchronisation. The ability of connected dynamic elements to synchronise their behaviour through interaction is ubiquitous (see [25] for a general introduction) and has profound implications for a wide variety of systems. We are particularly interested in the situation where the connected elements are oscillators [6], as their synchrony is observed in many settings, from the human heart [26] and brain [27], to insect locomotion [28] and novel forms of computation [29]. Previous work [30] has established a strong correlation between the connectivity of groups of nodes and the time required for oscillators to synchronise. However, given that full synchronisation does not (and, indeed, *should* not) occur in many networks (for example, the abnormal synchronisation in neurones is known to be a feature of epilepsy [24]) we are interested in the possible relationship between structure and dynamical behaviour for oscillator networks where the coupling between oscillators is weak enough and the connectivity in the graph is sparse enough, such that synchronisation does not occur. In this paper, we precisely address this question.

Network topology has a strong effect on the observed dynamics of oscillator networks [31]–[34]. Previous work has mainly focused on whether or not a network will synchronise, relating this to graphical measures such as the eigenvalues of the Laplacian [31] or clustering coefficients [35]. This work suggests that the ability of an oscillator network to synchronise is enhanced by homogeneity in the distribution of connections [31].

Many complex networks have been shown to demonstrate periodic dynamics. Neural systems, for example, display modes of oscillation at particular frequencies and this has in turn been linked to the hierarchical organisation of the brain network itself [36].

In coupled oscillator networks with all-to-all coupling, oscillating waves of synchronization have been observed in systems with bimodal and trimodal frequencies [37], [38] and in systems of interacting populations of oscillators [39]. Such oscillations may also be observed in globally coupled oscillators, where there is both an excitatory and inhibitory component to the interactions, as observed in [40], [41]. However, in each of these cases the global oscillations are in some way attributable to the individual nodes in the network and not to the network structure itself.

In this paper we show how the community structure of a complex network may actively drive periodic dynamics and that such periodic dynamics occur in real world networks. The remainder of this paper describes our methodology in detail, showing how a simple model system is capable of a variety of dynamical behaviours. We then give the results of experimental investigations into the effect of network topology on oscillatory dynamics and how the latter may be used to detect the former. In particular, we demonstrate how our methodology may be applied to two real world networks. We conclude with a discussion and suggestions for future work.

## Methods

In order to rigorously establish the relationship between network structure and dynamics, we require a model system that is broadly applicable, but which supports a wide range of dynamical behaviours. We also need to be able to measure the global network dynamics in a way that readily admits analysis. The well-established *Kuramoto model* [5], [42], [43] meets all of these requirements and is widely used in related work [44]–[47].

The model describes a system of coupled oscillators described by ordinary differential equations (ODEs) where interaction terms between oscillators are connected according to the specific network topology:
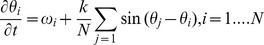
(1)where 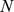 is the number of nodes in the network, 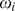 is the natural frequency of oscillator 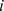, 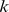 is the coupling strength between connected oscillators and 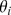 is some oscillatory phase 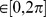.

This original model of Kuramoto assumes mean-field interactions. In the absence of any external noise, the global dynamics are determined by the coupling strength 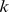, the distribution of natural frequencies 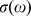 and the connectivity within the underlying network. In general, the coupling strength 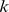 acts to synchronise the oscillators, the wider the distribution of 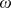, the harder it is for the oscillators to synchronise and higher connectivity within the graph also serves to cause the oscillators to synchronise (i.e. all to all coupling will synchronise more easily than sparsely coupled networks).

Many variations of the original Kuramoto model have been developed; of particular interest is the introduction of a *phase lag*, 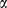, between oscillators, which can give rise to so-called *chimera states* [48]–[50]. These occur when oscillators form into clusters, some of which are synchronised and some of which are desynchronised. Chimera states are inherently interesting, because they describe a situation in which a collection of identical oscillators splits into two domains, one coherent and the other incoherent. As Abrams and Strogatz [48] observe, “Nothing like this has ever been seen for identical oscillators.”

Chimera states can arise as a direct result of network topology; specifically, the existence of community structure [51]. The observations we describe in this paper, although in many respects similar to such Chimera states in that global observations can be directly attributed to topology, are significantly different.

Motivated, in part, by the realisation that many naturally-occurring networks have complex topologies, recent studies have been extended to systems where the pattern of connections is local but not necessarily regular [52]. Due to the complexity of the analysis, further assumptions have generally been introduced. For example, it is usually assumed that the oscillators are identical. Obviously, therefore, in the absence of disorder, (i.e. if 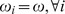) there is only one attractor of the dynamics: the fully synchronised regime, where 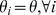. This scenario suggests that, starting from random initial conditions, a complex network with a non-trivial connectivity pattern will exhibit the following behaviour: first, the highly interconnected units that form local clusters will synchronise; second, in a sequential process, increasingly large synchronised spatial structures will emerge, until, finally, the whole population is synchronised [30]. However, for many dynamical complex networks, synchronisation is neither realised nor desirable. In these instances, weakly coupled oscillators may display partial synchronisation or clustering, but not full synchronisation. More formally, Equation 1 can give rise to a variety of dynamical behaviours. For strongly coupled networks (those with high connectivity and coupling strength 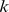) the phases of all oscillators quickly synchronise. With weak coupling, the oscillators appear to move randomly. Between these regimes, we observe *partial* synchronisation, where some oscillators are synchronised and others form clusters, but no global synchronisation is evident.

We use a global order parameter [47]:
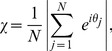
(2)as a measure of coherence over the entire network. This is the average phase of all oscillators within the network; for fully synchronised networks, 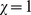; for networks where the phases of all oscillators are equally distributed around 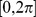, 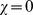 and for all other states, 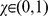. In what follows, we use the global order parameter to investigate the effect of network topology on synchronisation.

## Results

We now present the results of our experimental investigations. The over-arching aim is to show how global oscillatory behaviour may be related directly to the community structure of the underlying complex network.

### Artificial networks

**Figure 1:**
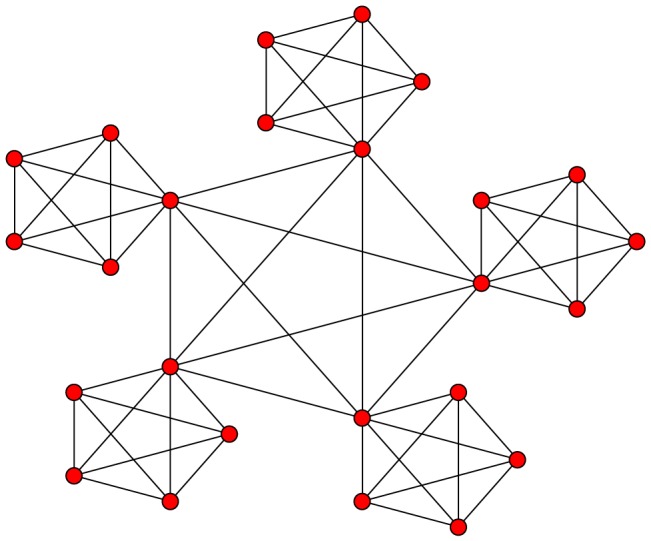
Example graph with community structure (one level of hierarchy). For such a network there exist parameter regimes where the smaller, globally connected sub-graphs may synchronise but the network as a whole does not (partial synchronisation or clustering).

**Figure 2:**
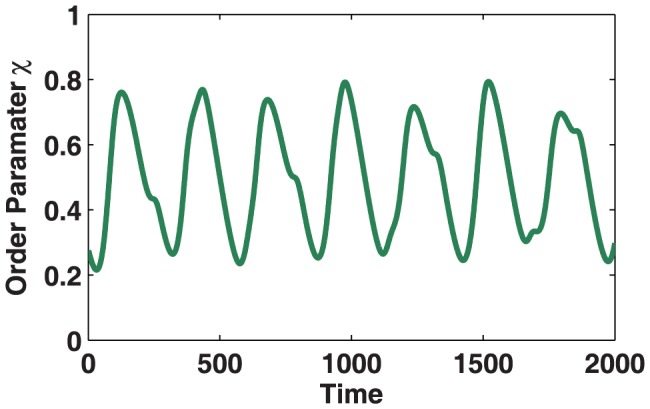
Kuramoto simulation for the network in Figure 1. Time series for order parameter, 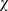, showing oscillatory dynamics for a network of Kuramoto oscillators coupled as in Figure 1. The coupling strength 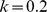 and the frequencies are normally distributed with standard deviation 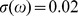. Note, for such parameter values it is possible to observe full synchronisation or oscillating dynamics as shown above depending on the individual frequencies of the oscillators. The example demonstrated here, although fairly typical, is not the only observable dynamics for such a network.

We first study two classes of graph; those with and those without any community structure. For example, consider the typical community structured graph in Figure 1. Given weak coupling, the dynamics of such a graph allow for the possibility of synchronisation within the smaller globally connected clusters, while the entire graph remains only partially synchronised. As such, any global measure of synchronisation appears to oscillate (Figure 2) the oscillation being dependent upon the differences in the frequencies of oscillations between each of the clusters.

Figure 2 shows the order parameter oscillating between relatively low levels of synchronisation and almost full synchronisation. We emphasise, though, that the internal frequencies of the oscillators, 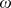, have been specifically selected in order to demonstrate such dynamics and that this will not occur in all cases. In graphs without any community structure, we fail to observe any discernible oscillation above that of the natural frequency of the oscillators.

**Figure 3:**
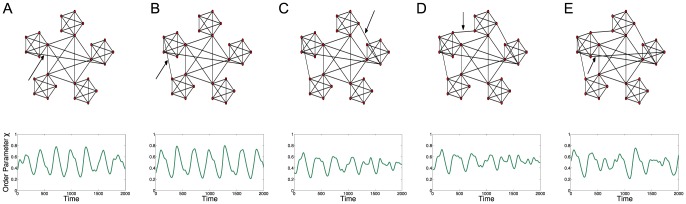
Kuramoto simulation for increasingly pertubed hierarchical network. Time series of global order parameter, 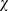, for increasingly perturbed hierarchical network of Kuramoto oscillators. New links are highlighted by arrows, demonstrating the robustness of dynamics to symmetry breaking, with 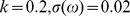. Each simulation uses the same initial conditions and oscillator frequencies as in Figure 2. Similar observations occur whether the simulations are conducted as individual runs (as shown here) or with the network structure being perturbed as the simulation is performed. Note: Although the time series for **C** and **D** appear very similar they are simulations from their respective graphs.

In order to demonstrate that the oscillating dynamics shown above are not simply an artefact of network symmetry, we perturb the original network by repeatedly adding random connections. Figure 3 demonstrates the structural stability of the modal dynamics when the network structure is no longer symmetric, but the community structure is retained.

Figure 3 demonstrates that the global oscillations observed are not due to symmetry of the graph structure. Although the asymmetric graphs no longer produce strong regular oscillations, the dynamics are not significantly affected by symmetry-breaking through the addition of connections. For this particular graph, it is possible to add a further 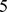 connections before the onset of global synchronisation.

**Figure 4:**
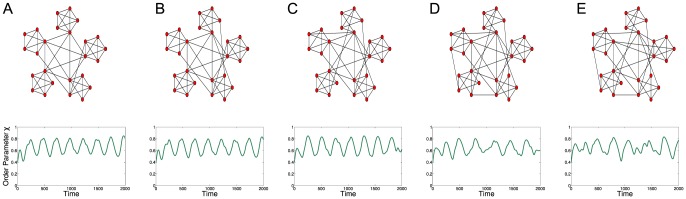
Kuramoto simulations for rewired hierarchical networks. Time series for global order parameter, 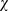, for various networks of coupled Kuramoto oscillators. Each network has been rewired using the Xswap rewiring algorithm which maintains the degree of each node. Two pairs of edges have been rewired from one graph to the next from **A** through to **E** and that the oscillating behaviour begins to break down as the hierarchical structure is decreased. 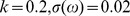. Again the same initial conditions and internal oscillator frequencies are used as in Figure 2.

Figure 4 shows another example of network rewiring, in this case using the Xswap algorithm [53], in which the network is randomised, with the degree of each node remaining constant. This is achieved by randomly selecting a pair of edges in the network, 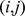 and 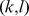. If 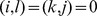 then 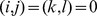 and 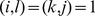. It should be noted that in the unperturbed network the nodes are self connected, so on some iterations these edges are swapped. The oscillations break down as the network is randomised demonstrating that it is the overall graphical structure that causes this behaviour.

To further develop the study of non-symmetric networks, we consider a large, idealised network of oscillators arranged such that three highly coupled sub-networks of oscillators are connected via a sparse network of random connections. We report the results of simulations for subgraphs of 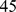 oscillators with approximately 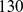 connections within each cluster.

**Figure 5:**
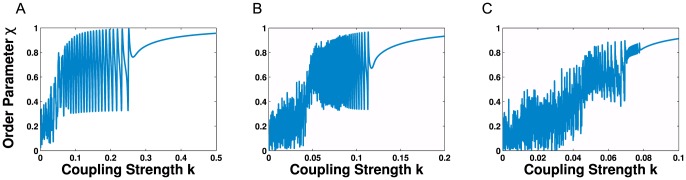
Coupling strength bifurcations for order parameter 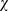 for networks of clustered random networks. Each network contains 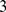 clusters of 45 randomly connected nodes with approximately 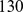 connections in each cluster. Here the frequencies are normally distributed with 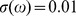. (**A**) 50 additional random connections over the whole network; (**B**) 100 additional connections; (**C**) 150 additional connections. Note: The oscillatory regions indicate the parameter regimes where oscillatory behaviour will be observed.

We first investigate the effect of varying coupling strength, 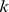, using standard bifurcation techniques. Figure 5 shows typical one parameter bifurcation diagrams of the global order parameter, 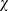, as 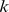 is increased from an initial value of 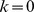 to 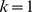. Here, the initial phases of the oscillators are drawn from a uniform distribution, 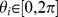. At each iteration of the simulation the value of 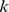 is increased in small increments, typically of around 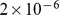 and we show bifurcations using 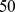, 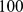 and 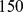 random additional connections (see Figure 5).

**Figure 6:**
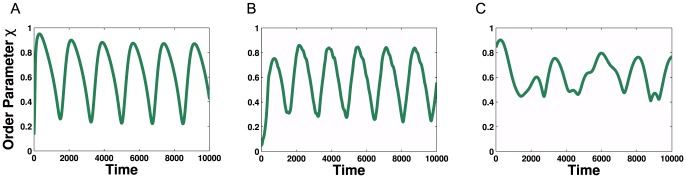
Kuramoto simulations for networks of clustered random networks. Time series for global order parameter, with 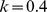 (**A**), 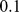 (**B**), and 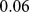 (**C**) showing multi-modal dynamics. The simulations are for networks described in Figure 5 and the parameter values taken from the oscillating regions.

In common with networks lacking community structure, these networks synchronise above a critical coupling strength; for small values of coupling strength, the oscillators are incoherent. In the first example there exists a specific region for 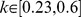 for which the order parameter, 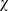, appears to oscillate between the ordered and disordered state. Figure 6 shows the time series of the order parameter for the three networks described above, with a distribution of internal frequencies of 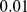 and respective coupling strengths of 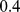 (**A**), 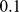 (**B**) and 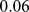 (**C**).

**Figure 7:**
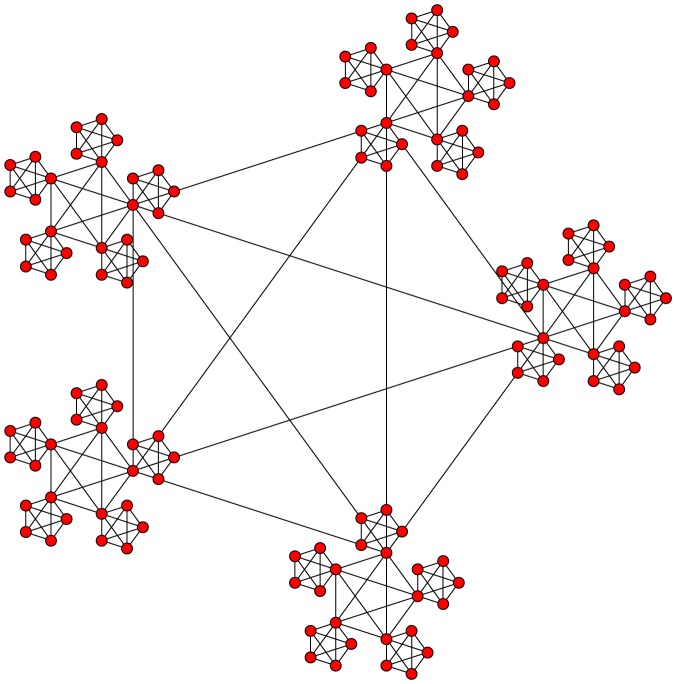
Example graph with community structure (two levels of hierarchy). For such a network there exist parameter regimes where the smaller, globally connected sub-graphs may synchronise but the network as a whole does not (partial synchronisation or clustering).

**Figure 8:**
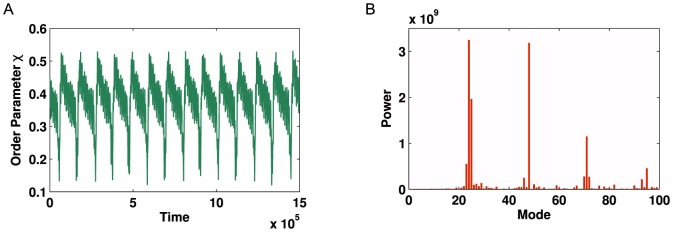
Kuramoto simulation and corresponding Fourier spectrum for the network shown in Figure 7. (**A**) Time series of global order parameter 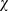 for a network with two levels of hierarchy with 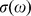 = 0.0012 and 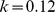. (**B**) Fourier spectrum for the signal in **A** demonstrating the modes of oscillation in the signal. Two strong peaks can be seen (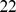 and 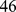) followed by their respective echoes (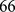 and 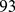).

We now consider a more complex network, which displays an additional level of hierarchy (Figure 7). For optimised parameter values of 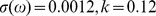 we observe multi-modal oscillations of the global order parameter, 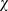, within a range of 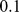 to 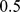. A Fourier spectrum of this time series demonstrates two modes of oscillation, at modes 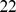 and 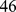, with strong echoes at modes 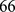 and 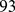 (Figure 8). The relationship between these oscillating modes strongly mirrors the graphical structure of the network, in that the two levels of hierarchy cause a bimodal oscillation and therefore two peaks in the Fourier spectrum.

### ‘Real world’ networks

In the previous section, we established the feasibility of using a global order measure to detect community structure in artificial networks. We now validate this approach against two classes of ‘real world’ network, both of which present examples that may or may not possess community structure.

In order to provide a metric for comparison, we use the standard measure of 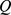 modularity [54]. The measure 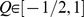 gives a sense of community structure and is defined as the proportion of the edges that fall within any cluster, minus the *expected* proportion if such edges were distributed at random. Other metrics for determining such modularity have also been proposed (see [55], for example); however we use the most well known (the MATLAB program to calculate 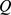 modularity was downloaded from VisualConnectome [56]).

### Human metabolic network

The metabolic network of a cell or microorganism describes the connections between various cellular processes that are essential for sustaining function [4]. Metabolic networks often exhibit strong community structure [57]–[59] and existing examples are usually examples of *pseudo-hierarchical* networks, in that their structure is not fully hierarchical [60]. In this Section we use our method to correctly identify community structure in metabolic networks.

We use metabolic pathway networks in SBML format [61], taken from the BiGG database [62]. These are imported to MATLAB using libSBML [63]. In this analysis, the *Homo Sapiens Recon 1* (human) metabolic network is used, as this is perhaps the most interesting example available. Similar results have been observed on other metabolic networks formulated in a similar manner.

**Figure 9:**
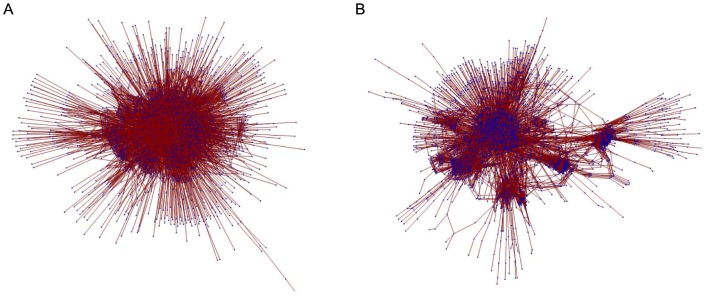
Graphical representations of two versions of the same human metabolic network. (**A**) Non-partitioned representation of human metabolic network. (**B**) Partitioned representation of human metabolic network in which the network is partitioned into sub-cellular networks. The Mathematica spring algorithm is used to display the network structures, it is apparent that the two versions have a very different structure.

In order to establish a relationship between community structure and dynamics, we consider two versions of this network. The first comprises the global connectivity matrix of all chemical reactants in the cell, a connection being present if two or more components are involved in a known reaction (we exclude water and ATP, as these occur in almost all reactions). The second formulation of the metabolic network partitions reactions into sub-cellular networks, each representing different regions of the cell (nucleus, golgi bodies, etc.) which are connected in turn by reactions. Graphical representations of these networks are shown in Figure 9.

From a graph theoretical perspective, these two networks are very similar. Standard graph metrics such as the clustering coefficient, mean and maximum path length do not distinguish between the two. Furthermore, the eigenvalue spectrum (as described in [30]) also shows no discernible difference.

The main difference between these two networks lies in the values for 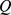 modularity, with the compartmentalised version having a value of 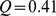 and the non-compartmentalised version having a value of 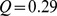. Due to the higher modularity of the compartmentalised version, we would expect to see regular oscillations in this representation.

**Figure 10:**
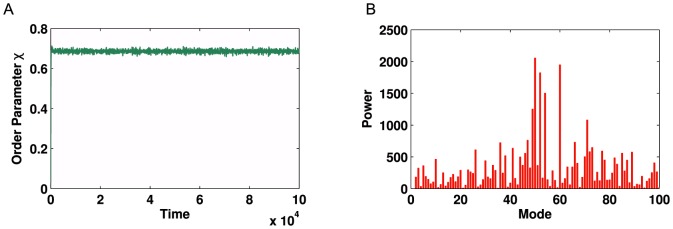
Kuramoto simulation and corresponding Fourier spectrum for the unpartitioned human metabolic network. (**A**) Time series of global order parameter 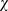 for the network shown in Figure 9 **A** with 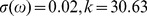. As no region of oscillation was found in the bifurcations for this network, parameter values were set to the same as those for the partitioned network, for the purposes of comparison. (**B**) Corresponding Fourier spectrum showing no strong peaks due to the signal not showing oscillatory behaviour.

**Figure 11:**
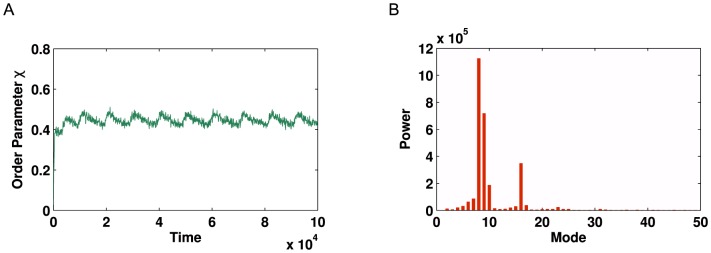
Kuramoto simulation and corresponding Fourier spectrum for the partitioned human metabolic network. (**A**) Time series of global order parameter 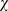 for the network shown in Figure 9 (**B** with 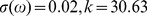. These variables were optimised to obtain a strong oscillatory dynamic. **B**) Corresponding Fourier spectrum showing a strong peak in the Fourier transform at mode = 7, followed by an echo at mode = 15, demonstrating the oscillatory behaviour of the signal.

Simulations for optimised coupling strengths and frequency distributions are conducted on both forms of the metabolic network. For the non-partitioned network, we fail to observe multi-modal oscillations in the global order parameter. However, for the partitioned network we observe strong modal dynamics (See Figures 10 and 11 for a comparison) which is consistent with the results for 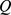 modularity. This demonstrates that our method of community detection is a viable method for use on complex real-world networks, where the underlying structure is not as regular as those formed using generative models.

### Transport networks

**Figure 12:**
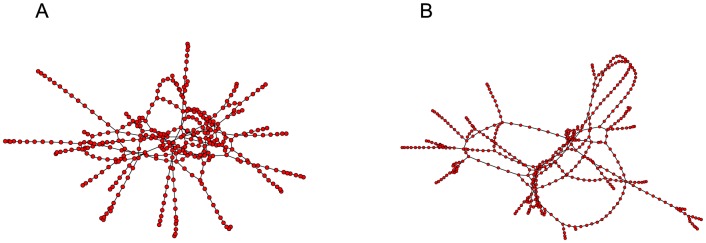
Graphical representations of two underground railway networks. The London Underground (**A**) and New York Metro (**B**) networks, represented as non spatially-arranged graphs (i.e. they represent station *connectivity*, rather than the actual geographical locations of stations). Note the presence of two central clusters in the New York graph, which represent the concentration of stations in South Manhattan and Brooklyn. Both of these networks representations were generated using the Mathematica spring algorithm.

We now investigate a completely different type of network; those describing mass transit systems in major cities. Specifically, we compare the network of the London Underground and the New York Subway systems, as both are large enough to be interesting, but they have very different underlying geographical structures. In particular, stations on the London Underground are more evenly distributed than in New York, where the presence of islands in the geography of the city gives rise to clusters of stations, particularly in South Manhattan and Brooklyn (Figure 12). Taking the 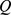 modularity of both of these networks gives London a value of 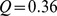 and New York a value of 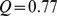. From this, we predict that our method will generate a regular oscillating pattern for New York, but not for London. The London underground and New York Subway maps were taken from the ‘Transport For London’ [64] and the ‘Metropolitan Transportation Authority’ [65] websites respectively. Using these maps we constructed, by hand, adjacency matrices in which stations are represented by nodes, with an edge connecting pairs of nodes if there exists a direct line between stations.

Structurally, these networks are significantly different from the previous examples. Notably, there exist many long chains, the overall graph connectivity is low and there exists very few ‘small world’ effects. As such, we are confident that these networks present a novel challenge, over and above that offered by both the artificially-generated networks and the metabolic networks.

**Figure 13:**
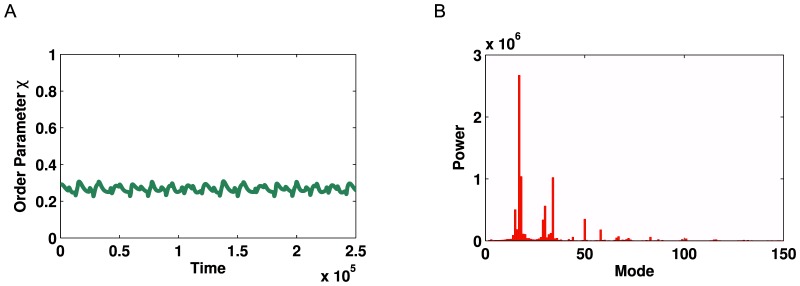
Kuramoto simulation and corresponding Fourier spectrum for the London Underground. (**A**) Time series of global order parameter 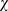 for the network shown in Figure 12 **A** with 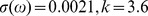. These values were chosen to maximise the oscillatory behaviour. (**B**) Corresponding Fourier spectrum for signal in **A**.

As before, we run numerical simulations in order to optimise model parameters, in an attempt to maximise any oscillatory dynamics. On the London network, we observe a small amount of oscillatory behaviour, although the amplitude of such oscillation is small - the maximum observed oscillation has an amplitude of 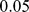. The resulting Fourier spectrum has a peak strength of 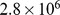 (Figure 13).

**Figure 14:**
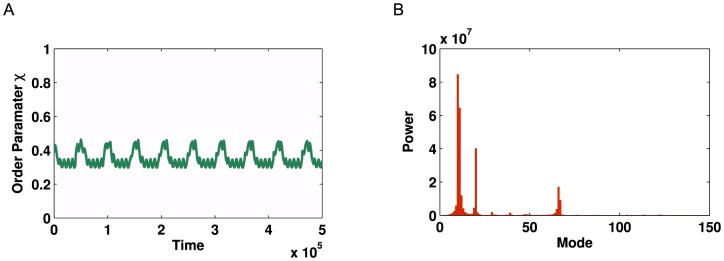
Kuramoto simulation and corresponding Fourier spectrum for the New York Metro. (**A**) Time series of global order parameter 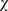 for the network shown in Figure 12 **B** with 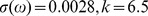. These values were chosen to maximise the oscillatory behaviour. (**B**) Corresponding Fourier spectrum for signal in **A**.

On the other hand, experiments on the New York network yield a *significantly* more pronounced oscillation, which displays very strong periodicity. The primary oscillatory mode has a strength of 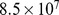 - and a strong echo. A second oscillatory mode is also observed (Figure 14).

In order to demonstrate that this oscillating behaviour is indeed caused by the underlying hierarchy of the network, the New York subway network was rewired using the Xswap algorithm previously described. We observe that as the network is rewired and the modularity reduced to below 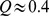, oscillations no longer occur. As the Xswap algorithm maintains the degree distribution of the network but reduces modularity, this precisely demonstrates that modularity directly causes the oscillations in the order parameter of the phase.

## Discussion

In this paper, we have demonstrated a robust and structurally stable relationship between form and function in complex networks whereby global oscillations are shown to be a factor of network topology. We observe modal oscillations in a measure of global synchronization which can be directly related to the community structure of the network itself.

By applying the method to two types of real world networks - whereby examples exist with significantly different community structures but with similar underlying topology, we show that this method also works on realistic, more irregular structures. We demonstrate the breakdown in oscillatory behaviour when networks are rewired (with the degree of each node remaining constant). This confirms that network modularity drives oscillations, as reducing the degree of modularity causes these oscillations to break down. We should note, however, that for the real world examples given, the underlying dynamics of the nodes on the network (chemical reactions and subway trains) are considerably more complex than the simple Kuramoto oscillators used to demonstrate the principle. As such, it is not possible to directly attribute any observed oscillatory dynamics in such systems to the network structure alone.

Many real world networks (e.g. transport, the brain) are examples of pseudo-hierarchical networks, in that their structure is not fully hierarchical [60]. In the particular example of the brain, for instance, multi-modal oscillations (observed as Gamma 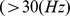 Beta 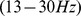 and Alpha 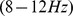 waves etc in EEG measurements) may attribute to structural hierarchies in the neural connectivity. As such, for systems where the dynamics of the individual elements of a complex network are known to be unimodal and the interactions between them are likewise, global observations of oscillatory behaviour may give some indication as to underlying structures and network connectivity, yielding novel methods of community detection.
